# P-1799. Impact of coronavirus disease 2019 on infectious disease treatment and infection control: Multicenter study in Japan

**DOI:** 10.1093/ofid/ofae631.1962

**Published:** 2025-01-29

**Authors:** Hiroshi Kakeya, Shigeki Kakuno, Waki Imoto, Ayumi Shintani

**Affiliations:** Osaka Metropolitan University, Osaka, Osaka, Japan; Osaka Metropolitan University Graduate School of Medicine, Osaka, Osaka, Japan; Osaka Metropolitan University, Osaka, Osaka, Japan; Osaka Metropolitan University Graduate School of Medicine, Osaka, Osaka, Japan

## Abstract

**Background:**

The coronavirus disease 2019 (COVID-19) pandemic impacted infection control practices such as hand hygiene and antimicrobial use, but its specific effects remain unclear. Thus, in this multicenter study, we aimed to determine the pandemic's effect on these factors by comparing pre- and post-COVID-19 periods.

(A) The number of inpatients by month during the study period. (B) The amount of hand sanitizer used per inpatient by month during the study period.

**Figure:**
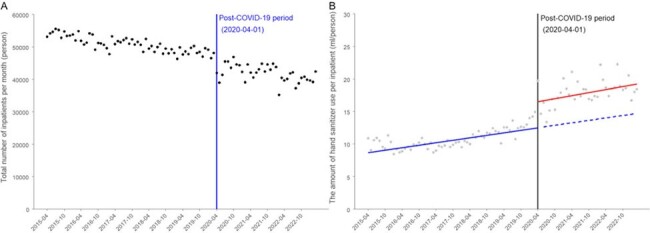

The two solid lines show the regression lines in the pre- and post-COVID-19 periods. The dashed line in the post-COVID-19 period is an extension of the regression line in the pre-COVID-19 period.

**Methods:**

Data on hand sanitizer use, antimicrobial use, positive blood cultures, and antimicrobial-resistant (AMR) organism detections were collected monthly from April 2015 to March 2023 at four Japanese hospitals. The pre-COVID-19 period data (April 2015 to March 2020) were compared to the post-COVID-19 period data (April 2020 to March 2023).

**Results:**

Compared to pre-COVID-19, hand sanitizer use per inpatient and antimicrobial use, particularly broad-spectrum antimicrobials, increased significantly post-COVID-19. The proportion of positive blood cultures increased, but AMR organism detections remained unchanged, suggesting increased contamination rather than infection.

**Conclusion:**

Ongoing antimicrobial use monitoring, hand hygiene education, and promotion of effective infection control and antimicrobial stewardship by infection control teams are crucial for prevention of future pandemics.

(A) Total DOT (combined with all antimicrobials extracted in this study) by month. (B–F) DOT of individual antimicrobial agents by month.

**Figure:**
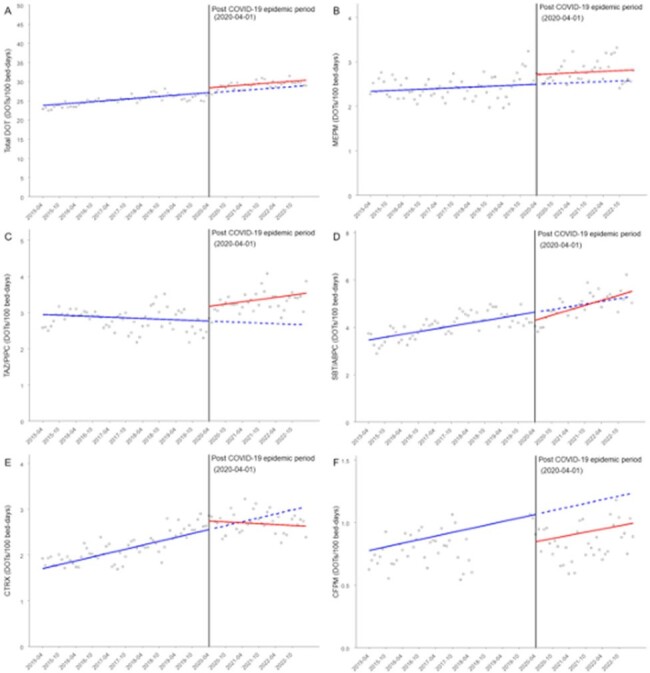

The two solid lines show the regression lines in the pre- and post-COVID-19 periods. Other antimicrobial agents are shown in Appendix Figure A.1.

Abbreviations: CFPM, cefepime; CI, confidence interval; CTRX, ceftriaxone; DOT, days of therapy; MEPM, meropenem; SBT/ABPC, sulbactam/ampicillin; TAZ/PIPC, tazobactam/piperacillin.

**Disclosures:**

**Hiroshi Kakeya, MD, PhD**, Asahikasei Phrma: Honoraria|GSK: Honoraria|MSD: Honoraria|Pfizer: Honoraria|Shionogi: Honoraria|Sumitomo Parma: Honoraria

